# A Multi-Sensorial Hybrid Control for Robotic Manipulation in Human-Robot Workspaces

**DOI:** 10.3390/s111009839

**Published:** 2011-10-20

**Authors:** Jorge Pomares, Ivan Perea, Gabriel J. García, Carlos A. Jara, Juan A. Corrales, Fernando Torres

**Affiliations:** Department of Physics, System Engineering and Signal Theory, University of Alicante, San Vicente del Raspeig, Alicante 03690, Spain; E-Mails: ivan.perea@ua.es (I.P.); gjgg@ua.es (G.J.G.); carlos.jara@ua.es (C.A.J.); jcorrales@ua.es (J.A.C.); Fernando.Torres@ua.es (F.T.)

**Keywords:** direct visual servo, human-robot collaboration, visual servoing, tactile control

## Abstract

Autonomous manipulation in semi-structured environments where human operators can interact is an increasingly common task in robotic applications. This paper describes an intelligent multi-sensorial approach that solves this issue by providing a multi-robotic platform with a high degree of autonomy and the capability to perform complex tasks. The proposed sensorial system is composed of a hybrid visual servo control to efficiently guide the robot towards the object to be manipulated, an inertial motion capture system and an indoor localization system to avoid possible collisions between human operators and robots working in the same workspace, and a tactile sensor algorithm to correctly manipulate the object. The proposed controller employs the whole multi-sensorial system and combines the measurements of each one of the used sensors during two different phases considered in the robot task: a first phase where the robot approaches the object to be grasped, and a second phase of manipulation of the object. In both phases, the unexpected presence of humans is taken into account. This paper also presents the successful results obtained in several experimental setups which verify the validity of the proposed approach.

## 1. Introduction

The development of flexible manipulation tasks by robotic manipulators in semi-structured environments with human operators presents an important challenge for the implementation of robot controllers. These robot controllers should perform context-aware tasks so that the robots’ trajectories are adapted to the changes in the environment. This context-awareness can be achieved by the combination of the information registered by several sensors. These sensors should be able to track not only the objects to be manipulated, but also the human operators who work in the same workspace. This paper proposes a multi-sensorial system composed of a camera (which is used to guide the robotic system towards the object to be manipulated), a human tracking system (which combines an inertial motion capture system and an indoor localization system to localize human operators) and several tactile sensors (which are used to execute the object manipulation tasks).

**Figure 1.**
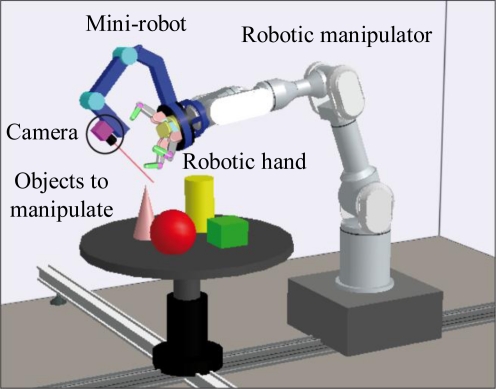
Multi-robot system composed of the robotic manipulator and the coupled mini-robot with a camera in an eye-in-hand configuration.

Different techniques have been implemented in order to interpret the information from these sensors for their application in the robot controller. Visual servoing techniques are usually applied to guide robots using information registered by cameras. However, robotic systems based on visual servoing suffer from occlusions, particularly in manipulation tasks where the robotic tool may block the line of sight between the camera and the object to be manipulated. In order to overcome this drawback, this paper proposes to use an additional mini-robot, which is installed at the end-effector of the robotic manipulator, as shown in Figure 1. The camera is installed at the end of this mini-robot, so that the camera can be moved independently to avoid occlusions caused by the robotic manipulator. This camera can be used to control the movements of both robots by considering its view of the object to be manipulated. On the one hand, the mini-robot is guided by employing a direct visual servoing control, which obtains the forces and torques to be applied to each joint of the robot in order to perform the desired task. This robot controller only takes into account the visual information of the object obtained from the camera of the mini-robot and does not consider the position of the human operator. This paper extends the previous direct visual control with a novel hybrid approach which uses the human-robot distance computed by the human tracking system in order to avoid collisions between any human who may enter the workspace and the mini-robot. On the other hand, the robotic manipulator is controlled by a Reference Virtual Camera (RVC) which simulates the use of an eye-in-hand camera from the images registered by the camera of the mini-robot.

In order to implement the manipulation of the object, this paper proposes the use of a robotic hand installed at the end-effector of the robotic manipulator (see Figure 1). This paper presents a novel tactile controller which takes into account the pressure information obtained from several tactile sensors installed over the fingers of this robotic hand. This tactile controller guarantees that a stable grasp of the object is kept while the fingers of the hand are moved to drive the object to a desired configuration.

This multi-sensorial system considers that the execution of the manipulation tasks is divided into two main phases (see Table 1): the first phase involves the robot approaching towards the object to be manipulated, and the second phase constitutes the manipulation of this object. During the first phase, the trajectory of the robotic manipulator is controlled by the RVC in order to move the robotic hand installed at its end-effector towards the object. A 3D model of the object is known. Meanwhile, the trajectory of the mini-robot is obtained by the direct hybrid controller. In this phase the robotic hand does not perform any movement because it is away from the object to be manipulated. During the second phase, the robotic manipulator does not move because it is already at the manipulation position and thus, the robotic hand performs the tactile control in order to execute the manipulation task. The trajectory of the mini-robot continues to be obtained by the direct hybrid controller since the human operator may enter the environment in any phase of the task.

This paper is organized as follows. Section 2 discusses the research works related to the approach presented in the paper. Section 3 describes the proposed hybrid controller. The particular control system developed to guide the mini-robot is detailed in Section 4. Section 5 expands the system proposed to position the robotic manipulator using the Reference Virtual Camera. The phase of autonomous manipulation of an object is described in Section 6. Section 7 shows different experiments to validate the proposed methods. Finally, the most important conclusions are discussed in Section 8.

## 2. Related Work

In an autonomous robotic manipulation task, the first topic that must be resolved is the positioning of the robot manipulator. In order to increase the safety and precision of the robotic task, visual servoing systems are usually employed to guide the movement of the robotic system using visual information [1]. Nevertheless, when a visually guided robot performs a manipulation task, the image features acquired from the sensors can be occluded by the robot tool, or by the own object geometry. These occlusions can appear not only in an eye-in-hand configuration (the camera is located at the end-effector of the robot), but also in an eye-to-hand approach (the camera observes the end-effector and the object). Therefore, the problem of occlusions is an important topic of research in visual servoing systems. The use of more than one camera can decrease the probability of an occlusion event during the visual servoing task [2]. In [3], the problem is tackled by introducing a second camera in order to have the benefits of both configurations: eye-in-hand and eye-to-hand. The eye-to-hand camera is positioned over a mobile robot that observes the manipulator (which has an eye-in-hand configuration). The same strategy is employed in [4], where both camera configurations are fused to improve the visual servoing task. In order to improve the manipulation tasks while guiding the robot manipulator using a visual servoing scheme, a similar approach to [3] is proposed in this paper. The main drawback in this type of solutions is that the occlusions are not completely solved despite the use of multiple cameras. However, in this paper only one camera is employed to improve the scene visibility. The approach proposed in order to solve the problem of occlusions involves the building of a mini-robot. This mini-robot has a camera located at its end-effector with the aim of obtaining a suitable viewing point for helping the robotic manipulator to manipulate the objects correctly. Therefore, contrary to the other mentioned approaches, only one camera is employed to improve the scene visibility.

Classical image-based visual servoing systems assume that the robot is a perfect positioning device. This type of control does not take into account the system dynamics, which is not suitable when the robot executes fast and/or accurate movements. As mentioned in [5], until the mid 90s, few proposed visual-based controllers took into account the non-linear dynamic robot-arm model. During the last 15 years, the research trend in this field has continued to be the same: controllers are designed with the assumption that the robot is a perfect positioning device without dynamics. By means of direct visual servo, the internal control loop of servo motors is removed, so that the visual servo control has to stabilize the robot. One of the first research works about direct visual servo control was the one developed in [6]. The controller proposed by Miyazaki and Masutani was based on the transpose Jacobian approach, which is a method implemented for the first time by Takegaki and Arimoto [7]. However, in this approach, the visual system was modelled as a simple rotation transformation, without taking into account the robot translation. Kelly and Márquez [8] implemented a more accurate camera-robot system model than the one proposed in [6]. This method solves the problem of the need to obtain the intrinsic parameters of the camera, but it requires accurate of its orientation. This problem is solved in [5], where the controller proposed in [8] is improved to consider the generated uncertainty for the camera orientation and thus, local asymptotic stability is achieved. During the first decade of the 21st century, adaptive control theory has been studied in depth, which allows solving the errors of the dynamic parameters estimation. This way, Zergeroglu *et al.* designed an adaptive controller which takes into account the estimation uncertainty of the camera-robot parameters [9]. In 2008, Wu and Li developed another adaptive controller which estimates the extrinsic and intrinsic parameters of the camera by considering a dynamic model of the robot [10]. In this field, the research of Wang *et al.* [11] is worth mentioning, in which the authors solve the visual control of a three d.o.f. robot by using an adaptive algorithm that updates, in each iteration, the distance value between the camera and the object. Thereby, this method obtains a distance-independent interaction matrix. Only a few works about direct visual servo include an image-based visual control approach, and neither of them permits to divide the space using a hybrid approach as presented in this paper.

Hybrid control involves the simultaneous control of separate directions with different sensors. Thus, hybrid visual/force control involves the simultaneous control of separate directions by vision and force [12,13], which is an extension of hybrid position/force control [14]. Hybrid control schemes have been widely developed using visual servoing systems: not only for combining visual and force sensors like in [12,13], but also for combining position-based and image-based visual servoing [15], or for integrating reinforcement learning with visual servoing [16]. The proposal in this paper is based on the hybrid control approximation. In order to assure the safety of a human operator who is working in a robotic cell, the mini-robot directions are partitioned. Hence, a direct visual servoing system controls the principal task of positioning, whereas the distance between the human and the robot controls the secondary task of human safety.

In regard to the manipulation phase, the robotic hand installed at the end-effector of the robotic manipulator executes a specific trajectory of its fingers in order to move the manipulated object to a desired configuration. In order to compute and execute this trajectory, a manipulation planner should be implemented by considering three different types of constraints [17]: fingers’ kinematics constraints, contact maintenance constraints and equilibrium constraints. The first type of constraints verifies that the fingers’ trajectories computed by the planner are kinematically feasible; in other words, the required movements of the fingers should be within the kinematic limits of their joints. The second type of constraints guarantees that the contact between the surfaces of the fingers and the object is maintained during the entire manipulation task. The third type of constraints verifies that the fingers apply a minimum contact force to the object in order to keep the grasp stable and thus undesired sliding between their surfaces is avoided. Previous research works propose different planners in order to solve this robotic manipulation problem [18]. Some of them (such as [19] and [20]) are only based on the geometric relations between the fingers and the object so that they resolve the kinematic and contact maintenance constraints but they do not consider the contact forces which are required to fulfill the equilibrium constraints. Other planners are based on punctual contact models with friction based on force closure, which guarantee theoretically that a specific grasp is able to counteract any external force applied to the object. These theoretical models are usually implemented as inequalities between the components of the friction forces which are solved by linear/quadratic programming (such as in [21] and [22]) or by conditions which are verified for each intermediate grasp of the object to be manipulated (such as in [23] and [24]). These planners use approximations where the surfaces of the fingers and/or the object are simplified in order to assume punctual contacts. This simplification involves that these planners are only applied in simple simulations and they are never applied in real manipulation tasks where objects and fingers with complex surfaces are used. In order to overcome this limitation, this paper proposes a novel tactile control algorithm that verifies that a robotic hand can perform a manipulation task of an object with arbitrary shapes by taking into consideration the contact pressures registered by tactile sensors installed over its fingers. This tactile control algorithm receives as input a list of finger joint angles which are obtained off-line by the geometric planner described in [25]. This planner considers the kinematic and contact maintenance constraints of the manipulation task in order to obtain a possible sequence of fingers movements which drive the object to the desired configuration. Afterwards, the proposed tactile control is applied in order to execute the planned fingers movements with the real hand. This tactile control guarantees that the fingers apply a minimum contact force to the object in order to avoid grasp instability caused by the loss of contacts between the object and the fingers. Thereby, the tactile control verifies that the equilibrium constraints are verified during the real execution of the robotic manipulation.

## 3. Visual Hybrid Controller

This section describes the hybrid controller proposed for the mini-robot guidance. Section 3.1 describes the concept of hybrid tasks. The primary task is defined as a visual servoing task to be performed by the robot. The secondary task is another sensor based task which will be used for avoiding collisions between the robot and the human operator. Furthermore, in Section 3.2 the hybrid controller is extended to direct control in order to improve the system behavior. Finally, Section 4 describes how this controller is applied for the guidance of the mini-robot.

### 3.1. Hybrid Tasks Definition

In image-based visual servoing systems, the control is directly carried out in the image space. Therefore, the controller input is a comparison between the observed image features **s** and the desired ones. The reference or desired features, s*, are expressed as features observed by the computer vision system in the desired location (*i.e.*, points, lines, circles, corners, *etc.*). The vision system is located in the feedback of the control loop, and deals with the extraction of these features during the task, **s**. The controller compares the real features and the desired ones and executes the necessary control actions in order to achieve the position where s = s*.

This technique is usually implemented in an external loop that provides an input to the robot’s internal controller. This approach corresponds with the indirect visual servoing, but contrary to indirect visual servoing, in direct visual servoing the internal joint controller of the robot is replaced by the visual controller, which uses the vision system data directly to control and stabilize the robot. This last approach is employed to guide the mini-robot during the phases of approaching and manipulation. However, in order to avoid collision between the human and the robot, a new hybrid approach is presented in the following paragraphs.

A visual servoing task can be described by an m-dimensional image function, **e**_1_, which must be regulated to 0:

(1) $${\text{e}}_{1}=B(\text{s}*-\text{s})=B\cdot {\text{e}}_{s}$$

where **s** = (*f*_1_, *f*_2_, … *f*_k_) is a k × 1 vector containing k visual features observed at the current state (*f*_i_ *= (f*_ix_, *f*_iy_*)*), while s* = *(f*_1_*, *f*_2_*, … *f*_k_*) denotes the desired features (extracted at the desired location). The visual features depend on the robot pose, **s** = **s**(**r**(t)), where **r**(t) is the relative pose between the camera and the environment at time t. **B** is an m × k combination matrix that must be of full rank m ≤ k in order to produce the m independent components of **e**_1_. The aim of visual servoing is to regulate the task function **e**_1_ to 0 so that **e**_s_ = 0.

The task **e**_1_ controls m degrees of freedom from a total of n. When m < n, this means that a virtual link of class N is fulfilled so that m = n − N ≤ k. The used combination matrix **B** is:

(2) $$B={\mathrm{WL}}_{s}^{T}$$

where ${\text{L}}_{s}^{T}$ represents the transpose of the interaction matrix [1]. **W** is an m × n matrix of full rank m and has the same kernel that **L**_s_. The choice of **W** depends on the number of features k. For example, let us consider the following cases:

- Rank (**L**_s_) = m = k → **W** = **L**_s_. In this particular case, **B** = **I**_m_.
- Rank (**L**_s_) = m < k → the rows of **W** are the m vectors which form the base of the row space generated by **L**_s_.

In a general case, with k visual features **s** only m robot degrees of freedom (DOF) are constrained, m = n − N ≤ k. Therefore, two different tasks will be defined [26]:

- **e**_1_: primary task (see Equation (1)). This is the visual task which employs the desired visual features, s*, to guide the mini-robot.
- **e**_2_: secondary task. This task is expressed as a cost function to be minimized under the constraint that **e**_1_ is satisfied. Furthermore, when **e**_2_ = 0, **W**^+^**W** = **I**_n_. Therefore, if the secondary task is null, all the degrees of freedom are controlled by the primary task.

Considering the previous assumptions, the following task function, **e**, which depends on the primary **e**_1_ and the secondary **e**_2_ tasks, is defined:

(3) $$\text{e}=W^{+}{\text{e}}_{1}+\left(I_{n}-W^{+}W\right){\left(\frac{\partial {\text{e}}_{2}}{\partial \text{r}}\right)}^{T}=W^{+}B\left(\text{s}*-\text{s}\right)+\left(I_{n}-W^{+}W\right){\left(\frac{\partial {\text{e}}_{2}}{\partial \text{r}}\right)}^{T}$$

where $\frac{{\partial \text{e}}_{2}}{\partial \text{r}}$ is the gradient of **e**_2_ and **I**_n_ – **W**^+^**W** is a projection operator on the null space of **W**.

In order to completely define the task function, the value of **e**_2_ must be described. **e**_2_ is defined as a cost function which depends on the task performed by the robot (see Section 4).

### 3.2. Direct Hybrid Controller

Robot dynamics expresses the relationship between the forces acting on a robot mechanism and the accelerations produced:

(4) $$\text{M}\left(q\right)\ddot{q}+C\left(q,\dot{q}\right)\dot{q}+G\left(q\right)=\tau$$

In this equation, **q** ∈ ℜ^nxl^ are the joint positions, **q̇** ∈ ℜ^nxl^ represent the joint velocities and **q̈** ∈ ℜ^nxl^ are the joint accelerations. Furthermore, **M** ∈ ℜ^nxn^ is the symmetric positive definite manipulator inertia matrix, **C**(**q**, **q̇)q̇** ∈ ℜ^nxl^ is the vector of centripetal and Coriolis torques, **G** ∈ ℜ^nxl^ is the vector of gravitational torques and **τ** ∈ ℜ^nxl^ are the effective joint torques. The hybrid controller that computes the suitable joint torques to regulate the task function **e** to 0 (see Equation (3)) is given by:

(5) $$\tau =K_{P}J_{g}^{T}\text{e}+J_{g}^{T}W^{+}\frac{{\partial \hat{\text{e}}}_{1}}{\partial t}-J_{g}^{T}\left(I_{n}-W^{+}W\right)\frac{\partial Gr\left({\text{e}}_{2}\right)}{\partial t}-K_{v}\dot{q}+G\left(q\right)$$

where $\frac{\partial {\hat{\text{e}}}_{1}}{\partial t}$ and $\frac{\partial Gr\left({\text{e}}_{2}\right)}{\partial t}$ represent the variation on the primary task and the gradient of the secondary task due to a possible autonomous target motion. **K**_P_ ∈ ℜ^nxn^ is the square diagonal nonsingular proportional matrix gain and **K**_v_ ∈ ℜ^nxn^ is the derivative matrix gain. These constants are symmetric positive-defined matrices that are estimated by the user. **J** = **J**(**q**, **s**) is defined as the Jacobian matrix and it is computed according the following equation:

(6) $$J\left(q,\text{s}\right)={\text{L}}_{s}\left(\text{s}\right)\cdot J_{g}\left(q\right)$$

where **L**_s_ (**s**) is the previous defined interaction matrix. **J**g = **J**g (**q**) is the geometric Jacobian matrix of the robot which relates the joint velocities and the translational and angular velocities (ν_c_, ω_c_ respectively) with respect to the camera frame located at the robot end:

(7) $$\left[\begin{array}{c} v_{c}\\ \omega_{c} \end{array}\right]=J_{g}\left(q\right)\dot{q}$$

In order to apply the previous defined controller in the mini-robot, the following assumptions are considered:

1. The scene is static, so that $\frac{\partial {\hat{\text{e}}}_{1}}{\partial t}$ and $\frac{\partial Gr\left({\text{e}}_{2}\right)}{\partial t}$ are zero. In case of non-static scenes, our previous approach [27] can be applied in order to determine the estimation of the autonomous target motion.
2. There exists a robot joint configuration **q**_d_ for which the task function is zero (and it is an isolated solution).

Considering the previous assumptions, the closed loop behavior can be easily obtained as:

(8) $$\text{M}\left(q\right)\ddot{q}+C\left(q,\dot{q}\right)\dot{q}=K_{P}J_{g}^{T}\text{e}-K_{v}\dot{q}$$

From the previous equation, the value of **q̈** can be obtained:

(9) $$\ddot{q}=\text{M}{\left(q\right)}^{-1}\left[K_{P}J_{g}^{T}\text{e}-K_{v}\dot{q}-C\left(q,\dot{q}\right)\dot{q}\right]$$

In order to demonstrate the controller stability, the following locally positive defined candidate Lyapunov function is employed:

(10) $$V=\frac{1}{2}{\dot{q}}^{T}\text{M}\left(q\right)\dot{q}+\frac{1}{2}{\text{e}}_{s}^{T}K_{P}{\text{e}}_{s}$$

The time derivative of the previous Lyapunov function is:

(11) $$\dot{V}={\dot{q}}^{T}\text{M}\left(q\right)\ddot{q}+\frac{1}{2}{\dot{q}}^{T}\dot{\text{M}}\left(q\right)\dot{q}-{\text{e}}_{s}^{T}K_{P}{\dot{\text{e}}}_{s}$$

and considering **ė**_s_= **J** · **q̇** and Equation (9) the previous time derivative function is equal to:

(12) $$\begin{array}{l} \dot{V}={\dot{q}}^{T}\left[K_{P}J_{g}^{T}\text{e}-K_{v}\dot{q}-C\left(q,\dot{q}\right)\dot{q}\right]+\frac{1}{2}{\dot{q}}^{T}\dot{\text{M}}\left(q\right)\dot{q}-{\text{e}}_{s}^{T}K_{P}J\dot{q}=\\ ={\dot{q}}^{T}K_{P}J_{g}^{T}\text{e}-{\dot{q}}^{T}K_{v}\dot{q}-{\dot{q}}^{T}C\left(q,\dot{q}\right)\dot{q}+\frac{1}{2}{\dot{q}}^{T}\dot{\text{M}}\left(q\right)\dot{q}-{\text{e}}_{s}^{T}K_{P}J\dot{q} \end{array}$$

Furthermore, the time derivative of the inertia matrix, and the centripetal and Coriolis matrix satisfy:

(13) $${\dot{q}}^{T}\left[\frac{1}{2}\dot{\text{M}}\left(q\right)-C\left(q,\dot{q}\right)\dot{q}\right]\dot{q}=0$$

Finally, from Equations (3), (12) and (13) it can be concluded that:

(14) $$\dot{V}={\dot{q}}^{T}K_{P}J_{g}^{T}\left(W^{+}B{\text{e}}_{s}+\left(I_{n}-W^{+}W\right){\left(\frac{\partial {\text{e}}_{2}}{\partial \text{r}}\right)}^{T}\right)-{\dot{q}}^{T}K_{v}\dot{q}-{\text{e}}_{s}^{T}K_{P}J\dot{q}$$

If there is no human in the workspace, all the degrees of freedom are controlled by the primary task. In this case the visual task constrains all the n degrees of freedom and **W** = **I**_n_, which leads to **I**_n_ – **W**^+^**W** = **0**. With this assumption, Equation (14) is equal to:

(15) $$\dot{V}={\dot{q}}^{T}K_{P}J_{g}^{T}{\text{L}}_{s}^{T}{\text{e}}_{s}-{\dot{q}}^{T}K_{v}\dot{q}-{\text{e}}_{s}^{T}K_{P}J\dot{q}=-{\dot{q}}^{T}K_{v}\dot{q}$$

**K_v_** is a symmetric positive-defined matrix, consequently **V̇** is a globally negative semi-definited function. Therefore, the point ${\left[{\begin{array}{cc} {\text{q}}^{T} & \dot{\text{q}} \end{array}}^{T}\right]}^{T}={\left[\begin{array}{cc} {\text{q}}_{0}^{T} & 0 \end{array}\right]}^{T}$ is a stable equilibrium.

In order to study the asymptotic stability, the LaSalle theorem can be applied. To do that, the following assumptions are considered:

- As previously indicated, a static target is considered, therefore, the closed loop system is autonomous.
- The Jacobian, **J**, is considered as continuous differentiable with respect **q**, so that, the rank of **J** is n.

To probe the condition of the LaSalle theorem, first, the components of the state vector [**q q̇**]^T^ which makes **V̇** = 0 must be determined. In this case, **V̇** = 0 when **q̇** = 0. Considering **q̇** and **q̈** equal zero, from Equation (8) the following equation is obtained:

(16) $$0=K_{P}J_{g}^{T}\text{e}=K_{P}J_{g}^{T}{\text{L}}_{s}^{T}{\text{e}}_{s}=K_{P}J^{T}{\text{e}}_{s}$$

In order to assure that the equilibrium [**q q̇**]**^T^** = [**q**_d_ 0]**^T^** is stable, we must conclude that **q** = **q**_d_ from Equation (16). As it is described in [5,8], considering the previous mentioned assumptions, Equation (16) has an isolated solution at **q** = **q**_d_, therefore, the equilibrium is locally asymptotically stable.

When the human enters within the workspace, the secondary task must be minimized under the constraint **e**_1_ = 0. Therefore a Cartesian controller is applied to some degrees of freedom determined by **I**_n_ – **W**^+^**W**. More details about the stability of Cartesians controllers based on Jacobian transpose can be seen in [28].

## 4. Mini-Robot Controller

As previously indicated, the mini-robot has been constructed by the authors. Therefore, the dynamical behavior is well known. This aspect permits to apply the direct hybrid controller presented in Section 3.2 to guide the mini-robot. The primary and secondary tasks employed by this robot are the following:

- **e**_1_: the primary task is an image-based task (see Equation (1)).
- **e**_2_: the secondary task is employed to avoid collisions between the robot and human operators.

When the human operator enters within the workspace, the primary task does not constrain all the n robot DOF. In this way, the other motion components are redundant and can be used to perform the secondary task **e**_2_.

The main goal of the secondary task is to guarantee that the mini-robot does not collide with the human operator while it is performing its task. In [4], a cost function is employed to avoid occlusions. However, in this paper another secondary task is defined so that it reaches its maximum when the collision occurs. The employed cost function is equal to:

(17) $$e_{2}=\alpha \left(1-\frac{\mathrm{dist}}{\mathrm{dmin}}\right)$$

where *dmin* is an empirical threshold so that **e**_2_ = 0 if *dist* > *dmin*, and *α* is a positive gain to be adjusted.

Thereby, the threshold *dmin* represents the minimum distance which must be kept between the mini-robot and the human operator. While the human-robot distance *dist* is above this threshold, the task **e**_2_ is null. Otherwise, this task moves the mini-robot away from the human as safety behaviour.

The distance *dist* between the human operator and the mini-robot is computed in real-time during the execution of the manipulation task by the safety human-robot interaction system described in [29]. This system is composed of a human tracking system and a precise human-robot distance computation algorithm based on bounding volumes.

**Figure 2.**
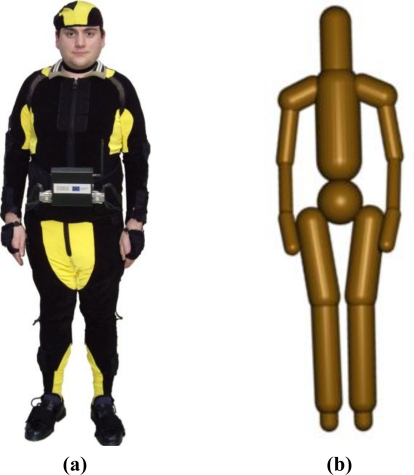
Safety system for human operators: **(a)** Human Tracking system; **(b)** Bounding volumes.

The human tracking system (see Figure 2(a)) is composed of an inertial motion capture system and an UWB (Ultra-WideBand) localization system [30]. The inertial motion capture system is based on a suit with 18 IMUs (Inertial Measurement Units) which register the relative rotations of all the limbs of the human body.

The UWB localization system is based on a small tag which is worn by the human operator and which emits UWB pulses to four sensors installed at fixed positions of the workspace. This UWB system computes the position of the tag and thus it obtains a good estimation of the global position of the human operator in the environment. The rotational measurements of the inertial system and the global position measurements from the UWB system are applied over a skeleton which represents the kinematic structure of the body of the human operator. Thereby, the resulting human tracking system is able to determine precisely the position of all the bones of the body of the human operator on real-time. The human-robot distance computation algorithm receives as input the previous skeleton of the human operator and the current joint values of the robots. Afterwards, it covers the skeleton of the human with a group of bounding volumes, Swept-Sphere Lines, (see Figure 2(b)) and it also covers each link of the mini-robot with these bounding volumes [31]. The positions of the links of the mini-robot are updated by applying forward kinematics to its joint values. Finally, the algorithm computes the distance between each pair of bounding volumes which cover the body of the human operator and the structure of the mini-robot. The distance *dist* applied for the control of the robot is established as the minimum distance value between all these pair-wise distance tests.

## 5. Robotic Manipulator Controller

### 5.1. Reference Virtual Camera

Only a vision-based task is employed for the robotic manipulator guidance. Therefore, the approach described in Section 3.1 can be used considering **W**^+^**W** = **I**_n_. In this case, only the task **e**_1_ is used. However, in order to guide the robotic manipulator, some modifications must be performed in the previous approach. The controller described in Section 3.1 considers that an eye-in-hand camera is employed. However, in our case the camera is located at the end of the mini-robot. To overcome this problem, a Reference Virtual Camera (RVC) located at the end of the robotic manipulator is used. The RVC is a virtual camera which will be employed to simulate the use of an eye-in-hand camera at the robotic manipulator.

Using the RVC, the image-based controller employed to guide the manipulator robot is:

(18) $$v^{\text{RVC}}=-\lambda {\text{L}}_{s}^{+}\left({\text{s}}_{u}-{\text{s}}_{u}*\right)$$

where **s**_u_ and **s**_u_* are the extracted and desired visual features considered by the RVC. In order to obtain these features, the approach described in the following paragraphs is employed.

First, we consider ${\text{M}}_{O}^{C}$ as extrinsic parameters of the real camera located at the end of the mini-robot (pose of the observed object frame with respect to the camera frame). Therefore, a 3D point of the object, ${\text{P}}_{P}^{O}$, can be expressed in the camera coordinate frame as:

(19) $${\text{P}}_{P}^{C}\left(x_{P}^{C},y_{P}^{C},z_{P}^{C}\right)={\text{M}}_{O}^{C}{\text{P}}_{P}^{O}$$

Considering a pin-hole camera projection model, the point ${\text{P}}_{P}^{C}$ with 3D coordinates relative to the camera reference frame is projected onto the image plane at the 2D point **p**. This point is computed from the camera focal length, f, as:

(20) $$\text{p}={\left(x,y\right)}^{T}={\left(f\frac{x_{P}^{C}}{z_{P}^{C}},f\frac{y_{P}^{C}}{z_{P}^{C}}\right)}^{T}$$

Finally, the units of Equation (20) specified in terms of metric units are scaled and transformed in pixel coordinates relative to the image reference frame, as:

(21) $$f=\left(f_{x},f_{y}\right)=\left(u_{0}+f_{u}x,v_{0}+f_{v}y\right)$$

where (f_u_, f_v_, u_0_, v_0_) are the camera intrinsic parameters. More specifically, (u_0_, v_0_) is the position of the optical center and (f_u_ = s_x_, f_v_ = s_y_) represent focal length in terms of pixel, where s_x_ and s_y_ are the scale factors relating pixels to distance.

**Figure 3.**
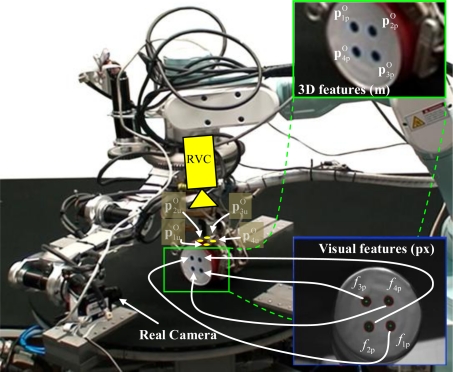
Reference Virtual Camera scheme.

In order to perform the robotic manipulator guidance using the RVC, the following steps have been implemented:

1. Considering **s** = (***f***_1p_, ***f***_2p_, …, ***f***_kp_,) as the extracted visual features by the camera located at the end of the mini-robot, the pose of the observed object frame with respect to the mini-robot camera ${\text{M}}_{O}^{\text{MC}}$ must be obtained (estimation of the camera extrinsic parameters). To determine the value of ${\text{M}}_{O}^{\text{MC}}$, an initial estimation of the previous pose is used ${\left({\text{M}}_{O}^{\text{MC}}\right)}_{1}$. A 3D model of the observed object is also known. This model stores the position of the characteristic points with respect to the object frame ${\text{P}}_{p}^{O}=\left({\text{p}}_{1\text{p}}^{O},{\text{p}}_{2p}^{O},...,{\text{p}}_{\text{kp}}^{O}\right)$ which will be extracted by the camera at the mini-robot. Furthermore, the model stores other characteristic points which will be employed to reconstruct the visual information captured by the RVC, ${\text{P}}_{u}^{O}=\left({\text{p}}_{1u}^{O},{\text{p}}_{2u}^{O},...,{\text{p}}_{\text{ku}}^{O}\right)$. Figure 3 represents the characteristic points ${\text{P}}_{\text{p}}^{\text{o}}$ extracted by the camera at the mini-robot and the points ${\text{P}}_{u}^{O}$ extracted by the RVC.
  To determine the value of ${\text{M}}_{O}^{\text{MC}}$, an additional controller is defined which progressively reduces the error indicated in Equation (22). This error represents the difference between the observed data, **s**, and **s**_p_. This last set of features is the position of the same features as **s** but computed by back-projection employing the current extrinsic parameters, ${\left({\text{M}}_{O}^{\text{MC}}\right)}_{1}$ Equations (19) and (21):
  (22) $${\text{e}}_{v}={\text{s}}_{p}-\text{s}$$
  The time derivative of **e**_v_ is equal to:
  23 $${\dot{\text{e}}}_{v}={\dot{\text{s}}}_{p}-\dot{\text{s}}=\frac{{\partial \text{s}}_{p}}{\partial \text{r}}\frac{\partial \text{r}}{\partial t}={\text{L}}_{s}\frac{\partial \text{r}}{\partial t}$$
  To make **e**_v_ decrease exponentially to 0, **ė**_v_ = –λ_2_**e**_v_, the following control action is obtained:
  24 $$\frac{\partial \text{r}}{\partial t}=-\lambda_{2}{\text{L}}_{s}^{+}{\text{e}}_{v}$$
  where λ_2_ is a positive control gain. $\frac{\partial \text{r}}{\partial \text{t}}$ represents the variation to be applied, at each iteration, to the extrinsic parameters, ${\left({\text{M}}_{O}^{\text{MC}}\right)}_{i}$. Once the error is cancelled, the value of ${\left({\text{M}}_{O}^{\text{MC}}\right)}_{i}$ will be equal the extrinsic parameters of the camera located at the end of the mini-robot.
2. From ${\text{M}}_{O}^{\text{MC}}$ and the mini-robot kinematics ( ${\text{M}}_{\text{MC}}^{\text{RVC}}$), the homogeneous transformation matrix between object O and the RVC can easily be obtained as ${\text{M}}_{O}^{\text{RVC}}={\text{M}}_{\text{MC}}^{\text{RVC}}\cdot {\text{M}}_{O}^{\text{MC}}$.
3. From the object 3D model it is possible to obtain ${\text{P}}_{u}^{O}$ (3D pose of the characteristic points with respect to the frame O, see Figure 3). This last set of features is different of the object features extracted by the mini-robot. Using this information and the matrix ${\text{M}}_{O}^{\text{RVC}}$, the pose of the characteristic points with respect to RVC is equal to ${\text{P}}_{u}^{\text{RVC}}={\text{M}}_{O}^{\text{RVC}}\cdot {\text{P}}_{u}^{O}$. From the previous pose and using Equations (20) and (21), the value of the visual features in pixel coordinates relative to the image reference frame **s**_u_ can be obtained. These features will be the extracted features used at each iteration of the visual servoing task.
4. As a classical image-based visual servoing is performed, the desired features are extracted by using a learning stage in which the robotic manipulator is moved to the desired location. In this stage, the pose of the mini-robot is unknown (the mini-robot can be located at any pose). Therefore, to learn the desired features (**s**_u_*) the steps 1–3 must be performed but considering the robotic manipulator located at the desired pose.

## 6. Robotic Manipulation Based on Tactile Control

As described in the Introduction section, while the robotic manipulator is approaching the object to be manipulated, the robotic hand installed at its end-effector is situated over this object. When the RVC control of the robotic manipulator stops since the approaching phase has finished, the manipulation task of the robotic hand is executed. First of all, an automatic closing process of the fingers of the hand is performed until the object is restrained by the fingers and its initial grasp is obtained. In order to calculate this initial configuration of the fingers, previous grasp synthesis algorithms could be applied [32]. Then, the in-hand manipulation task of the object is executed in order to drive the object to a desired configuration by controlled movements of the fingers. In order to develop this task, tactile information is needed. This information is obtained from several tactile sensors installed over the surfaces of the fingers of the hand. Each sensor is composed by an array of pressure-sensing elements (*i.e.*, tactels). Thereby, each sensor obtains an array of contact pressure values which correspond to the pressure applied to each tactel. This paper proposes a tactile control algorithm which takes into consideration the maximum pressure values from these tactile arrays in order to execute the movements of the fingers which are required to move the manipulated object.

This tactile control algorithm receives as input a list of finger movements (*i.e.*, a trajectory of finger joint angles) and executes them while verifying that each finger exerts a minimum contact pressure over the object so that the contact is not broken. The finger joint trajectory is computed by the geometric planner for robotic manipulation presented in [25] by considering the kinematic limits of the fingers and the geometric models of their surfaces (represented as triangle meshes). For each step of the previous finger joint trajectory, the tactile control algorithm is executed. Algorithm 1 shows the general pseudo-code of the developed tactile control algorithm.

**Algorithm 1.** Tactile Control Algorithm.

**Algorithm: Tactile Control Algorithm**	
**Input: *JointTrajectory[1..nSteps][1..nFingers]***, ***readjustThreshold[1..nFingers]***, ***safetyThres[1..nFingers]***	
**Implementation:**	
01:	**for***i* = 1..*nSteps*
02:	Execute Joint Angles of step *i* of the trajectory for all fingers: ***JointTrajectory[i][1..nFingers]***.
03:	**for***f* = 1..*nFingers*
04:	*numReadjust = 0*
05:	Get Maximum contact pressure *p* for finger *f* from its tactile sensor arrays.
06:	**if***p* < ***readjustThres[f]*** && *numReadjust* < NUM_MAX_READJUSTMENTS
07:	Increment joint angles of finger *f* if joint torques of *f* do not exceed maximum allowable torque
08:	*numReadjust = numReadjust+*1
10:	**goto** line 5
11:	**end if**
12:	**if***p* < ***safetyThres[f]*** && *numReadjust ==* NUM_MAX_READJUSTMENTS
13:	**return** // End manipulation task
14:	**end if**
15:	**end for**
16:	**end for**

First of all, this algorithm sends to all the fingers the commands which are required to move them to the joint angles stored in the trajectory from the geometric planner (line 2 of Algorithm 1). After the movements of the fingers, the tactile control algorithm obtains the maximum contact pressure which is applied by each finger to the object from its tactile sensors (line 5 of Algorithm 1) in order to compare it with two thresholds: the readjustment threshold and the safety threshold. This maximum contact pressure is firstly compared with the readjustment threshold. If the contact pressure is above the threshold, this means that the finger is applying sufficient pressure to the object and the following finger is verified. Otherwise, the tactile control algorithm executes a small increment of the joint angles of the finger (line 7 of Algorithm 1). This small increment supposes a readjustment of the finger where its position does not change (since the finger is blocked by the surface of the object) but its contact pressure over the object is increased (since the torques applied to the joints of the fingers are increased and thus the force applied by its fingertip). Then, the contact pressure of this readjusted finger is compared again with the readjustment threshold in order to determine if more readjustments are necessary. Before executing each readjustment, the algorithm verifies that the maximum allowable joint torques of each finger are not exceeded.

This iterative process can end in two different situations: either the contact pressure is increased by the successive readjustments over the readjustment threshold or a maximum number of readjustments is exceeded. This maximum number of readjustments is established in order to avoid infinite loops in the tactile control algorithm when the relative configuration between the object and the finger does not permit sufficient pressure increments to reach the desired threshold. When the maximum number of readjustments is exceeded, the contact pressure is compared with the second threshold, *i.e.*, the safety threshold. If it is above this threshold (line 12 of Algorithm 1), the algorithm continues and repeats the process with the following contacting finger. Otherwise, the manipulation task is stopped (line 13 of Algorithm 1) and the subsequent finger movements of the trajectory from the geometric planner are not performed since the contact between the finger and the object is too weak and more movements could create undesired sliding between their surfaces, which provokes grasp instability. If the maximum pressures of all the contacting fingers are over the corresponding safety thresholds, the following movement of the joint trajectory is executed. The process is repeated for the following step of the finger joint trajectory and it finishes when all the steps of the trajectory are executed. The readjustments of this algorithm involve an increase of the pressure of those contact points which are weak. Nevertheless, they do not involve an increase of the net force which is applied over the object and thus, undesired movements of the object are avoided. When a finger increases its contact pressure over the object, the forces transmitted to the other contacting fingers through the object are counteracted automatically by their motor controllers in order to keep their angular values.

The main contribution of the proposed tactile control algorithm is the use of real contact pressure measurements in order to guarantee that finger-object contacts are not broken. As stated in Section 2, previous robotic manipulation planners which consider contact pressures/forces are based on the force closure principle. These algorithms are based on applying restrictions over the shapes of the surfaces of the fingers and the object (such as spherical or parametric surfaces) and over the contacts between them (such as point contacts). These restrictions permit to efficiently solve the constraints established by force closure, but they limit considerably the types of fingers and objects that can be applied in manipulation tasks. The proposed tactile control algorithm overcomes these limitations since it does not need any supposition about the surfaces and the contacts. This algorithm only takes into consideration the contact pressure values registered by the tactile sensors installed over the fingers. Therefore, the proposed tactile control algorithm does not verify the force closure of the intermediate grasps during the manipulation task. Nevertheless, the omission of the force closure principle does not involve the generation of unstable grasps during the manipulation task because the algorithm is able to avoid them by stopping the manipulation process when the contact of one finger is too weak, which is an indication of an imminent undesired break of contact.

## 7. Experimental Results

In this section, three experiments are described in order to show the correct behaviour of the approaches described in the paper. As described previously, the experimental architecture consists of a multi-robot system composed of a robot manipulator and a mini-robot. A camera is located at the end-effector of the mini-robot, whereas the end-effector of the robot manipulator consists of a robotic hand with tactile sensors.

**Figure 4.**
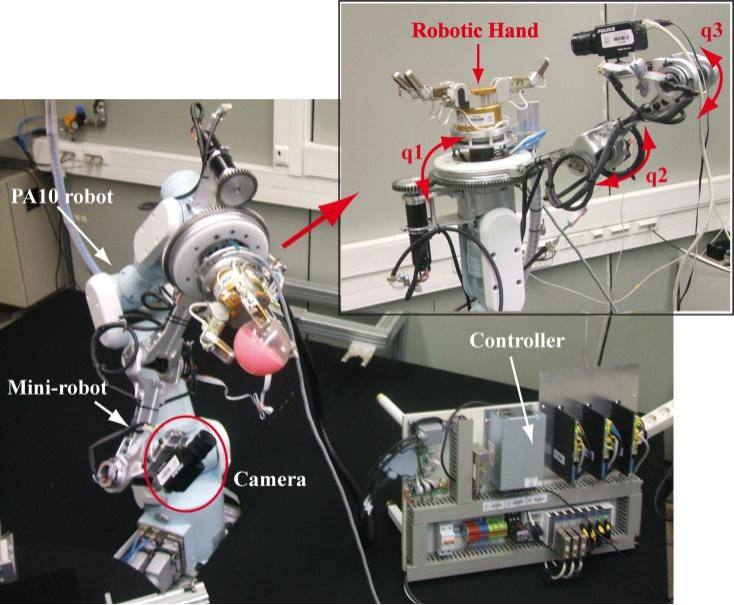
Multi-robot system setup.

The mini-robot (built with the aim of helping the robotic manipulator by increasing the visibility during the manipulation task) is a joint RRR robot with three DOF attached to the end of the robotic manipulator, a Mitsubishi PA-10 (see the multi-robot system setup in Figure 4). This joint structure has been successfully adapted at the end of the PA-10 without physical modifications in the latter. All the rotary joints of the mini-robot are independent of the robotic manipulator movement. The first rotary joint (q_1_) is used to place the mini-robot at any point around the manipulation task. The other two joints (q_2_, q_3_) move the camera on the point to achieve the objective and to acquire images without occlusions. To develop all the experiments, a Gigabit Ethernet TM6740GEV camera is mounted at the mini-robot end-effector. This camera acquires 200 images every second with a resolution of 640 × 480 px. The camera intrinsic parameters are (u_0_, v_0_) = (298, 225) px, and (f_u_, f_v_) = (1,082.3, 1,073.7) px. Finally, a three-fingered Barrett hand and seven PPS RoboTouch tactile sensors (two tactile sensors for each finger and one tactile sensor for the palm) have been employed to perform the manipulation tasks.

In the first experiment, the robotic manipulator describes a trajectory in order to approach the robotic hand towards the object to be grasped. Although the proposed system is able to react to the presence of a human operator, during this experiment, the human operator does not interact with the robot and all the DOF of the mini-robot are controlled by the primary task of the hybrid controller. To test the correct behavior of the safety strategy, in the second experiment, the robotic manipulator performs the same trajectory. However, during the approaching phase the human enters within the robot workspace. Finally, in the third experiment the manipulation phase is tested, presenting a multi-sensorial scheme to perform manipulation tasks in a robotic cell.

The execution time of the tactile control algorithm is limited by the sampling rate of the tactile sensors because the computational cost of the algorithm is negligible since it is based on comparisons with constant thresholds. Therefore, it is executed with a frequency of 30 Hz (*i.e.*, 33 ms for each execution step).

### 7.1. Experiment 1

**Figure 5.**
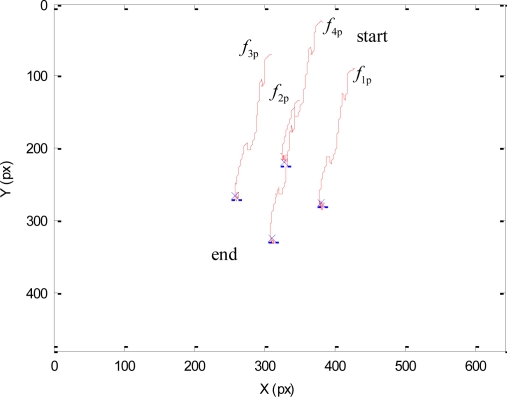
Trajectory of the visual features extracted by the camera at the end of the mini-robot during the first experiment.

**Figure 6.**
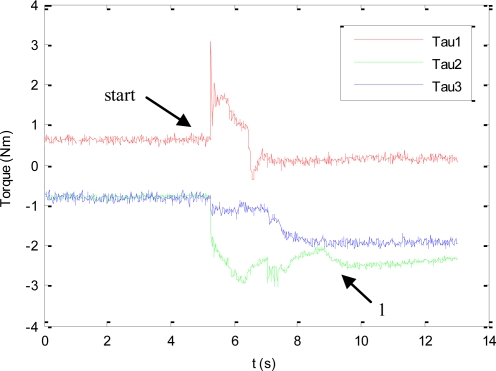
Torques employed to guide the mini-robot during the first experiment.

**Figure 7.**
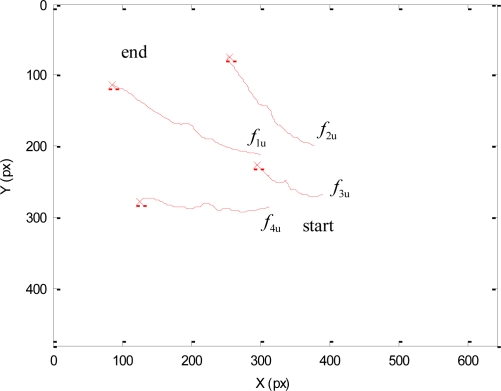
Image trajectory described by the robotic manipulator using the RVC.

As previously described, during this experiment the human will not enter within the robot workspace and all the DOF of the mini-robot are controlled by the vision-based task. The main objective of this experiment is to test the direct visual servoing scheme proposed in Section 3.2, as well as the RVC scheme proposed in Section 5 to guide the robotic manipulator, without the constraint of the human presence imposed by the hybrid controller. To do so, in this experiment, the matrix **W**^+^**W** = **I**_n_ in Equation (3). The desired features employed by the mini-robot controller in the primary task are s* = (f_1_*, f_2_*, f_3_*, f_4_*) = ((324,207), (377,272), (307,322), (257,259)) in pixels. Using these values, the image trajectories described by the features extracted by the mini-robot are shown in Figure 5. To perform this trajectory, the torques generated by the proposed controller are shown in Figure 6. In this figure, the label “1” indicates the moment when the mini-robot reaches the desired features, (**s** = s*), but the experiment continues until the robotic manipulator achieves the corresponding desired features, **s**_u_*. The desired features required to guide the robotic manipulator are computed as described in Section 5. For this experiment, the set of visual features obtained at the goal position of the RVC is **s**_u_* = ((254,79), (293,226), (131,274), (93,116)) in pixels. During this last phase of the experiment, the mini-robot compensates the robotic manipulator motion in order to maintain **s** = s*. The trajectory of the virtual visual features extracted by the RVC during the robotic manipulator guidance is represented in Figure 7. It is possible to observe that the desired features are achieved.

### 7.2. Experiment 2

Initially, both the robotic manipulator and the mini-robot desired features are the same as indicated in the Experiment 1. The main difference with respect to the previous experiment is the presence of the human operator. When the human enters within the workspace, the robotic manipulator is near its desired location and this robot only performs a decrease in depth. At this moment, the goal of the mini-robot is to observe the y-coordinate of *f*_1p_ at position 272, *i.e*., *f*_1py_* = 272 px. Only one DOF is required to perform the image-based task **e**_1_ of the mini-robot direct hybrid controller. This DOF compensates the motion of the robotic manipulator. The other motion components will be used to carry out the secondary task. However, all the visual features are extracted to obtain the necessary information to guide the robotic manipulator.

**Figure 8.**
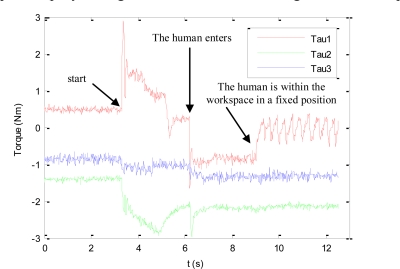
Torques employed to guide the mini-robot during the second experiment.

The torques applied during this second experiment are shown in Figure 8. At the beginning of this experiment, the human does not enter the workspace. Therefore, only the primary task is applied and the visual features extracted by the mini-robot are maintained at the desired location. When the human enters within the workspace, the cost function (17) increases and the secondary task is employed to avoid the collision.

**Figure 9.**
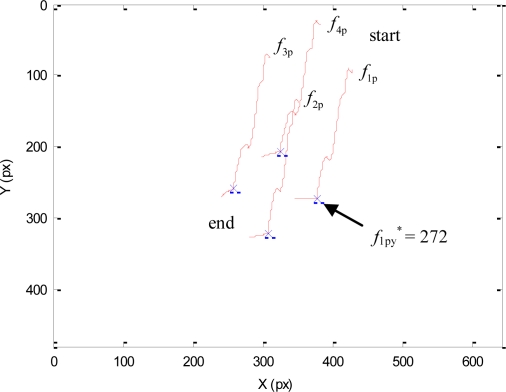
Trajectory of the visual features extracted by the camera at the end of the mini-robot during the second experiment.

Figure 9 represents the image trajectory described during this experiment. When the human operator enters in the workspace, the primary task compensates the decrease in depth of the robotic manipulator and the secondary task causes a small variation in the image features in order to avoid the collision (f_1py_* = 272 px). Once the human goes out of the robot workspace (dist > dmin), only the primary task is employed, which considers the desired features indicated in the Experiment 1 (for the sake of clarity this last phase is not represented in Figure 9).

**Figure 10.**
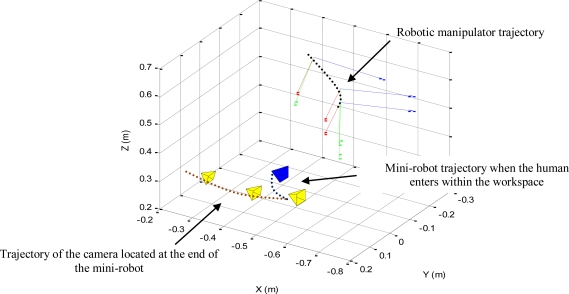
3D trajectory of both robots during the second experiment.

The 3D trajectories described by the robotic manipulator and the mini-robot in these experiments are represented in Figure 10.

The trajectory of the camera located at the end of the mini-robot during the first phase of the experiment is represented in yellow. The trajectory described due to the effect of the secondary task is represented in blue. A sampling of the 3D trajectory of the robotic manipulator is also shown (for each sample, the coordinate frame at the end of the robotic manipulator is represented). The correct positioning of both robots is achieved and the mini-robot does not collide with the human operator while it is performing its task.

### 7.3. Experiment 3

In order to develop the manipulation phase of robotic tasks, this paper proposes the use of a three-fingered Barrett hand installed at the end-effector of the robotic manipulator. This hand has seven tactile sensors installed over its fingers: one tactile sensor for each outer phalanx of each finger, one tactile sensor for each inner phalanx of each finger and a tactile sensor for the palm. The tactile sensors of the outer phalanxes are composed by an array of 22 tactels with an area of 36 mm^2^ per tactel. The tactile sensors of the inner phalanxes are composed by an array of 24 tactels with an area of 25 mm^2^ per tactel. The tactile sensor of the palm contains an array of 24 tactels with an area of 100 mm^2^ per tactel. All these tactels register pressure values in the range 0–140 kPa, with a sampling rate of 30 Hz and a sensibility of 0.7 kPa.

**Figure 11.**
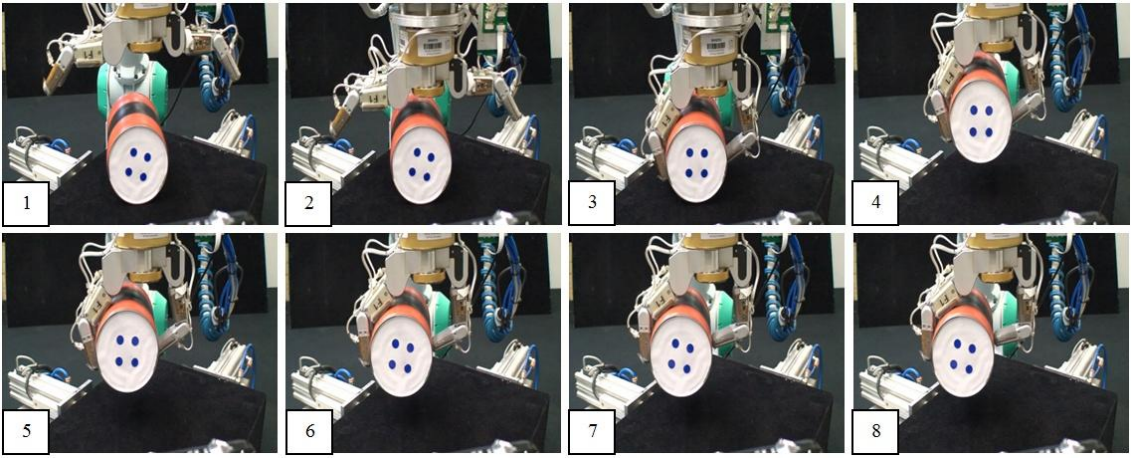
Sequence of frames of the manipulation phase of the robotic task.

Figure 11 depicts a manipulation task of a cylinder with the robotic hand employed in this paper. First of all, the robotic hand performs an automatic closing process of the fingers in order to establish the initial grasp of the object (as shown in frames 1–4 of Figure 11). Then, a joint trajectory of the fingers is obtained by the geometric planner of [25] in order to translate the cylinder in the perpendicular direction of the fingers. This planner is implemented in a software simulator [33]. Afterwards, the tactile control algorithm proposed in Section 6 is performed in order to execute the finger joint trajectory previously computed by the geometric planner. Frames 5–8 of Figure 11 show several of these finger movements generated by the tactile control algorithm.

**Figure 12.**
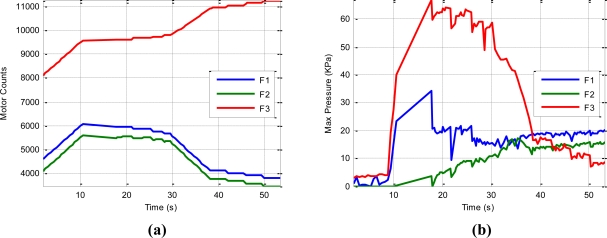
Evolution of fingers’ parameters during the execution of the manipulation task: **(a)** motor counts; and **(b)** maximum contact pressure.

Figure 12(a) depicts the evolution of the motor counts of each finger during the development of the task. These values are proportional to the joint angles of the corresponding finger because they are mechanically coupled and driven by a unique motor. The different slopes in this plot correspond to the execution of the finger movements calculated by the geometric planner. The horizontal lines in this plot correspond to the execution of finger readjustments by the tactile control algorithm since they suppose an increase in the contact pressure but without really moving the fingers because they are blocked by the surface of the object. These finger readjustments are executed when the maximum contact pressure of a finger is below the readjustment threshold: 10 kPa for fingers 1–2 and 20 kPa for finger 3. Figure 12(b) depicts the evolution of the maximum contact pressure of each finger. For instance, in the time interval t = [18 s, 30 s] several readjustments of finger 2 are performed because its pressure is below the readjustment threshold (10 kPa). These finger readjustments increase the contact pressure of finger 2 until it surpasses the readjustment threshold, as shown in instant t = 28 s of Figure 12(b). In the interval t = [30 s, 38 s] several finger movements without readjustments are performed since the contact pressure of all fingers are over their readjustment thresholds. In the interval t = [38 s, 53 s], several readjustments are necessary for finger 3 because its pressure is below its readjustment threshold (20 kPa).

Regardless of all these readjustments, the contact pressure of finger 3 decreases gradually. After executing the maximum number of readjustments, the manipulation task is stopped because the contact pressure is below the safety threshold (10 kPa). The end of the manipulation task is necessary because the surface of finger 3 is in contact with the object near to its edge so that further movements could provoke the breaking of the contact between finger 3 and the object and thus, grasp instability.

## 8. Conclusions

This paper has presented a multi-sensorial system which solves the problem of autonomous robotic manipulation in a human-robot workspace. The approach proposed is composed by the following sensors: a camera which is used to guide a multi-robotic system towards the object to be manipulated, a human tracking system which combines an inertial motion capture system and an indoor localization system to localize human operators, and several tactile sensors which are used to execute the manipulation task of the object.

The new controller shown in the paper is based on a hybrid control which combines the information from these sensors in order to solve the addressed problem. Firstly, in order to overcome the problem of occlusions in a manipulation task, an additional mini-robot has been built and installed at the end of the robotic manipulator. Thereby, the camera is installed at the end of this mini-robot to avoid occlusions which are caused by the robotic manipulator. The mini-robot is guided by using a novel hybrid direct visual servoing control which takes into account both the visual information of the object obtained from the camera and the position of the human operator within the workspace. The robotic manipulator is controlled by another approach called RVC, which simulates the use of an eye-in-hand camera from the images registered by the camera of the mini-robot. In addition, the paper has shown a novel tactile controller which takes into account the pressure information obtained from several tactile sensors installed over the fingers of the robotic hand. This tactile controller guarantees that a stable grasp of the object is kept while the fingers of the hand are moved to drive the object to a desired configuration. Finally, the paper has shown the results obtained in three experiments which validate the proposed multi-sensorial controller.

## Figures and Tables

**Table 1.** Phases and control used by each one of the robotic systems in the proposed approach.

**Phases**	**Robot Controller**	
Approaching	Robotic manipulator	*RVC*
	Mini-robot	*Direct Hybrid Controller*
	Robotic Hand	*No Control*
Manipulation	Robotic manipulator	*No Control*
	Mini-robot	*Direct Hybrid Controller*
	Robotic Hand	*Tactile control*
